# Uptake of core outcome sets by clinical trialists in China: a protocol

**DOI:** 10.12688/f1000research.139282.3

**Published:** 2024-07-17

**Authors:** Ruijin Qiu, Xiaodan Fan, Wenhui Wang, Mike Clarke, Zhuo Chen, Shuling Liu, Paula Williamson, Hongcai Shang

**Affiliations:** 1 Key Laboratory of Chinese Internal Medicine of Ministry of Education and Beijing, Beijing University of Chinese Medicine Affiliated Dongzhimen Hospital, Beijing, China; 2 University of Liverpool, Liverpool, England, UK; 3 College of Integrated Chinese and Western Medicine, Hunan University of Chinese Medicine, Changsha, China; 4 Queen's University Belfast, Belfast, Northern Ireland, UK

**Keywords:** Core outcome sets; clinical trialists; uptake

## Abstract

**Background:**

The concept of core outcome sets (COS) has been introduced in China for about 10 years. In recent years, some Chinese researchers also committed to developing COS, though the majority of COS are ongoing. However, there were more than 500 published COS for research in the COMET database by 2020. The extent of availability of COS for the top 25 diseases with the highest burden in China is unknown. In addition, the uptake of COS in clinical trials for these diseases is unknown, along with the knowledge, perceptions, and views of the clinical trialist community in China on the use of COS in relation to choosing outcomes for their research.

**Methods:**

The main burden of disease in China will be identified. Then we will search the COMET database to identify if there are ongoing or completed relevant COS research A COS published since 2012 would be preferred to one published before 2012 for the analysis of COS uptake if one meets the eligibility criteria. We will extract scopes of published eligible COS, including condition, population, interventions, and core outcomes. Then we will search the Chinese Clinical Trial Registry using disease names for each disease that has a published COS. We will assess the overlap in scope between clinical trials and COS. Then we will conduct an online survey and semi-structured interviews to identify the knowledge and perceptions of COS among primary investigators of included clinical trials.

**Discussion:**

This research will fill in gaps between COS and the burden of disease in China. Understanding clinical trialists’knowledge and perceptions of COS may help dissemination and application of COS in the future.

**Trial registration:**

This research is registered in Core Outcome Measures in Effectiveness: https://www.comet-initiative.org/Studies/Details/2563.

## 1. Background

A core outcome set (COS) is an agreed standardized set of outcomes that should be measured and reported, as a minimum, in all clinical trials in specific areas of health or health care. ^1^ The concept of COS was introduced in China in 2013. ^2^ Chinese researchers have registered more than 80 COS for research in the Core Outcome Measures in Effectiveness Trials (COMET) database by 2022.

A search of the COMET database shows that more than 500 completed COS studies have been published by 2021. The number of COS varies by disease category. The top five disease categories are cancer, rheumatology, neurology, heart and circulation, orthopaedics and trauma. ^3^ However, the number of candidates vary by disease category. In addition, there are more than one COS for some conditions, while no COS exists in some other important conditions. Whether there are gaps between COS research and the burden of disease in China remains unclear.

There are some benefits to using COS in clinical trials, which include improving the consistency of outcome reporting, increasing the relevance of outcomes measured to decision-makers, and helping to identify potential selective outcome reporting bias. However, previous research has shown that COS uptake is low in most research areas. ^4^^,^^5^ Research shows that clinical trialists’ awareness and understanding of COS may facilitate the use of COS. ^6^ However, Chinese clinical trialists’ awareness, understanding and using of COS are unknown.

The aims of this study are to examine: (1) whether the top 25 diseases with the highest burden in China have relevant COS; (2) the use of COS in clinical trials registered in the Chinese Clinical Trial Registry for these 25 diseases; and (3) views of these clinical trialists on the knowledge, perceptions, and use of COS in relation to choosing outcomes, as well as barriers and facilitators to uptake of COS.

## 2. Methods

### 2.1 Mapping of burden of disease in China to COS

***2.1.1 Identification of diseases for study***

The top 25 causes of disability-adjusted life-years (DALY) in China in 2019 have been identified from the tool of Global Burden of Disease (GBD). ^7^ Two researchers (RQ and XF) will also discuss subtypes of disease according to practice guidelines or textbook of internal medicine if necessary. The initial list of diseases for the study is shown in Table 1.

**Table 1.** The initial list of diseases for the study (ranked by number of disability-adjusted life-years).

Disease category	Burden of disease	Subtypes of disease
Neurology	Stroke	Ischemic stroke, intracerebral hemorrhage, subarachnoid hemorrhage
Heart & circulation	Ischaemic heart disease	Angina pectoris, acute coronary syndrome, myocardial infarction
Lungs & airways	Chronic obstructive pulmonary diseases	/
Cancer	Tracheal, bronchus, and lung cancer	/
Orthopaedics & trauma	Road injuries	/
Ear, nose, & throat	Age-related hearing loss	/
Orthopaedics & trauma	Low back pain	/
Endocrine & metabolic	Diabetes mellitus type 2	Diabetes mellitus type 2
Cancer	Stomach cancer	/
Neurology	Migraine	/
Mental disorders	Depressive disorders	Depression, dysthymia
Orthopaedics & trauma	Falls	
Neonatal	Neonatal disorders	Neonatal preterm birth Neonatal encephalopathy due birth asphyxia and trauma Neonatal sepsis and other neonatal infections Hemolytic disease and other neonatal jaundice Other neonatal disorders
Orthopaedics & trauma	Neck pain	
Cancer	Colon and rectum cancer	Colorectal cancer
Muscle disease	Other musculoskeletal disorders	Exclude: osteoarthritis, rheumatoid arthritis
Neurology	Alzheimer's disease and other dementias	Alzheimer’s disease Dementia
Kidney disease	Chronic kidney disease	Chronic kidney disease due to diabetes mellitus type 1 Chronic kidney disease due to diabetes mellitus type 2 Chronic kidney disease due to hypertension Chronic kidney disease due to glomerulonephritis Chronic kidney disease due to other and unspecified causes
Cancer	Esophageal cancer	/
Heart & circulation	Hypertensive heart disease	/
Cancer	Liver cancer	Liver cancer due to hepatitis B, liver cancer due to hepatitis C, liver cancer due to alcohol use, liver cancer due to NASH, liver cancer due to other causes
Infectious disease	Total burden related to hepatitis B	/
Sense organ diseases	Blindness and vision loss	Glaucoma, cataract, age-related macular degeneration, refraction disorders, near vision loss, other vision loss
Muscle disease	Osteoarthritis	/
Mental disorders	Anxiety disorders	/

***2.1.2 Identification of relevant COS***

We will manually search the COMET database and identify all COS for diseases/subtypes in the diseases list. We will identify if there are ongoing or completed relevant COS research. The study began on April 7 ^th^, 2023 and is ongoing.

***2.1.3 Inclusion criteria for COS***

(1) The scope appears relevant to a disease in the diseases list;
(2) COS for research should have included patient participants;

RQ will identify COS from the COMET database, and XF will check the results. Any discrepancies will be resolved by consensus discussion or by consulting a third reviewer (PW). RQ and PW will discuss all the COS for the 25 causes of DALY and determine which COS are eligible. The criterion related to the inclusion of patient participants follows previous work examining uptake of specific COS, and reflects the impact of patient participation on the choice of core outcomes. ^8^

The aim of COS is to promote a single unified approach to evaluating outcomes in a given condition throughout the world, however only 30% of COS published up to the end of 2021 have included participants from anywhere in Asia, ^8^ and the majority of COS developed in China are focused on traditional Chinese medicine (TCM). To explore whether the uptake of COS is influenced by whether it was developed by Chinese researchers, we will additionally include a COS published in a Chinese language journal if one meets the inclusion criteria above.

***2.1.4 Data extraction***

We will describe the number of ongoing and published COS for the highest burden of disease in China. Full texts will be retrieved for published COS only.

Previous COMET systematic reviews, and ongoing inclusion of published COS by COMET, will be used for details of published COS relevant to this work. Data extraction will include the intended use of recommendations, disease category, disease name, population characteristics (age, sex), interventions, geographical locations of COS developers, geographical locations of COS participants will be extracted, and whether patients participated.

***2.1.5 Data analysis***

Descriptive analysis will be used for general characteristics of COS.

### 2.2 Exploring uptake of COS in trialists in China

***2.2.1 Identification of clinical trials in the Chinese Clinical Trial Registry***

We will search the Chinese Clinical Trial Registry ( https://www.chictr.org.cn/historyversionpub.aspx) according to the disease name extracted for the published COS.

***2.2.2 Inclusion and exclusion criteria for clinical trials***

**Inclusion criteria for clinical trials:**

(1) Randomised and non-randomised clinical trials;
(2) The objectives of clinical trials are for disease therapy;
(3) The prospective registration time should be in 2021 and 2022.

**Exclusion criteria for clinical trials:** The clinical trials are to assess the mechanisms of effect of interventions.

If the disease name in the COS is broader, we will further search subtypes of disease. RQ and XF will discuss the list of subtypes of disease, any disagreement will be discussed with a third investigator (HS). XF, WW, ZC, and SL will search for clinical trials from the Chinese Clinical Trial Registry and apply the inclusion and exclusion criteria to identify the eligible clinical trials. RQ will check the results.

***2.2.3 Comparison of COS with clinical trials***

Inclusion criteria:

(1) An eligible COS exists published since 2012.
(2) The number of clinical trials with matching scope to an eligible COS is not less than 40.

A COS published since 2012 would be preferred to one published before 2012 for the analysis of COS uptake if one meets the eligibility criteria. A COS developed by Chinese researchers may be significant for Chinese clinical trialists. If there is an eligible COS developed by Chinese researchers, it will be included. If it does not meet the inclusion criteria for comparison of COS with clinical trials, then the prospective registration time for clinical trials will be expanded.

An online survey will be sent to trialists who registered clinical trials which are relevant to a specific COS. We anticipate a 50% non-response rate, thus if the number of trials is too small, it will be difficult to obtain a reasonable spread of perspectives from trialists about the use of the relevant COS. It was felt that responses from 20 trialists would be helpful based on a typical number of participants involved in qualitative research, thus requiring an anticipated 40 registered trials.

***2.2.4 Data extraction and analysis***

Data extraction for COS will include: author, title, publication time, condition, population, intervention, outcomes, and outcome measurement instruments (if applicable).

Contact details of trialists (including applicant’s name, the registration contact person’s email and telephone number, the primary investigator’s name, the primary investigator’s email and telephone number) will be extracted, because they will be invited to participate in the online survey. The other data extracted will include: title, the primary investigator’s institutions, study type, disease name, population, intervention, outcomes, and outcome measurement instruments.

For comparing trial outcomes and COS, only published COS will be used. If there are more than two COS for a specific disease, the differences in scope between these COS will be discussed by two reviewers. 

We will compare the overlap in scope between clinical trials and COS according to the framework used in previous research. ^9^ The framework is shown in Table 2.

**Table 2.** The framework for assessing overlap in scope between clinical trials and COS.

		Intervention			
		COS is narrow	Exact match	COS is broader	Different intervention
Population	COS is narrow	A	B	C	D
	Exact match	E	F	G	H
	COS is broader	I	J	K	L
	Different subgroup of the population	M	N	O	P

We will calculate the median percentages of outcomes in each clinical trial that were specific matches, general matches, and non-matches with outcomes in each relevant COS. We will calculate the proportion of outcomes that overlap between the COS and clinical trials for each disease.

### 2.3 Exploring awareness, barriers and facilitators to uptake COS by trialists

***2.3.1 Online survey***

**2.3.1.1 The process of online survey**

A survey will be sent to clinical trialists who have registered or completed a clinical trial that has overlapped scope between the trial and COS. We will identify the primary investigator’s name, email, and telephone number when we extract eligible clinical trials from the Chinese Clinical Trial Registry. We will send a Chinese translation of the COS deemed relevant to the trialists’ trials with questionnaire. When the primary investigators cannot be contacted via emails, the applicants for registration will be approached. We will examine trialists’ knowledge and awareness of COS. An online survey will be sent to clinical trialists, with one of three versions of the survey: 1) clinical trialists who report a full COS in their clinical trials; 2) clinical trialists who report only a few of outcomes in the COS; and 3) clinical trialists who do not report any outcome in the COS.

The survey will include both closed and open-ended questions. General information, such as gender, age, participants’ role (clinicians or researcher), specialty (traditional Chinese medicine, integrative medicine, or Western medicine), work experience, geographical area, professional qualification, and educational background will be collected. We will refer to the facilitators and barriers identified in previous research, including using of COS and clinical practice guidelines, ^10^^,^^11^ to develop open and closed questions to ask all participants. MK, PW, and RQ will discuss the questions in the questionnaires. The survey and other tools will be distributed into Chinese. RQ will translate tools into Chinese and the translation will then be sent to several researchers who have experience in COS research from a Chinese authors’ team and we will ask them to give comments. RQ, SL, ZC, and WW will discuss and determine the translation before distribution.

The structured questions in the survey are shown in extended data.

At the end of each survey, we will ask the trialists if they would like to attend a semi-structured interview.

**2.3.1.2 Data analysis**

Closed questions will be analyzed using descriptive statistics. Ratings for the barriers and facilitators to uptake will be summarized in terms of the median, range, and interquartile range. For open questions, thematic analysis will be used. All free text will be extracted in to a word document which RQ will read multiple times to organize the text into some initial themes and the author team will review the data and its assignment to the themes identified. The reason why the trialists used or did not use COS will be analyzed by content analysis. New suggested potential barriers and facilitators will be mapped to different themes.

***2.3.2 Semi-structured interviews***

**2.3.2.1 The process of semi-structured interviews**

We will invite trialists who agree to be interviewed to attend an in-person or online interview, if saturation is reached, the interviews will be completed. The COS publication, translated into Chinese, will be sent to them before the interview. The topic guide is as follows:

1. How many years of experience do you have in the design, management, or analysis clinical trials?
2. How many clinical trials did you participate in the design, management, or analysis?
3. When you design a clinical trial, how did you choose outcomes?
4. Do you know what is COS?
5. Are you familiar with COS?
6. Can you define COS?
7. How comfortable you are with English?
8. How did you become familiar with COS?
9. If there is a relevant COS in your research area, do you want to use it? Why?
10. Whether you know the COS that we sent you? Will you use it in your next trial?
11. What kind of facilitators and barriers for you to use a COS in clinical trial?
12. In your opinion, what should be done to improve the use of COS?

**2.3.2.2 Data analysis**

General characteristics of interviewees will be presented using descriptive methods. The trialist’s awareness of, and decisions to search for and use a COS will be described, and the reasons why trialists used or did not use COS will be analyzed by content analysis.

### 2.4 Dissemination

The finding of this research will be disseminated through conferences, peer-reviewed publication, and social media.

## 3. Discussion

COS have been introduced in China for about 10 years. Chinese trialists’ knowledge and awareness of using COS are important to COS developers in China. This study will provide information on the proportion of trialists in China who registered clinical trials in the Chinese Clinical Trial Registry in the areas with the highest burden of disease, and how the outcomes they chose compare to existing COS.

COS are very important for reducing heterogeneity of outcome reporting, potential bias, and research waste when they are used in clinical trials. There are hundreds of COS published in the world. Chinese researchers are also developing COS in some health areas, whether there is a gap between COS and the burden of disease in China remains unclear. This research will provide evidence relevant to COS researchers and clinical trialists.

## Ethics and consent

The project has been approved by the Ethics Committee of Dongzhimen Hospital, Beijing University of Chinese Medicine (2023DZMEC-175-01). The ethical approval agreed to not getting written informed consent from the participants, because they are clinical trialists.

**Trial registration:** This research is registered in Core Outcome Measures in Effectiveness: https://www.comet-initiative.org/Studies/Details/2563.

## Data Availability

No data are associated with this article. DANS-EASY; Questionnaire of trialists’ perceptions in COS. https://doi.org/10.17026/dans-ze4-tby3. ^12^ This project contains the following extended data:
• Questionnaire of trialists’ perceptions in COS(1).pdf. (English version of survey). Data are available under the terms of the Creative Commons Attribution 4.0 International license (CC-BY 4.0).
