# Supplementary material for: Uptake of core outcome sets by clinical trialists in China: a protocol
Source: F1000Res. 2024 Jul 17;12:1030. Originally published 2023 Aug 23. [Version 3] doi: 10.12688/f1000research.139282.3 (PMC10997984; doi:10.12688/f1000research.139282.3)
Supplement: Peer review history

# Reviewer report: Reviewer response for version 2

**DOI:** 10.5256/f1000research.164025.r260341

**Published:** 2024-04-05

**Authors:** Kim Thomas, Emma Campbell

**Affiliations:** 1 Centre of Evidence Based Dermatology, School of Medicine, University of Nottingham, Nottingham, UK

No further comments to make.

Is the study design appropriate for the research question?

Yes

Is the rationale for, and objectives of, the study clearly described?

Partly

Are sufficient details of the methods provided to allow replication by others?

Partly

Are the datasets clearly presented in a useable and accessible format?

Not applicable

Reviewer Expertise:

Applied health research, core outcome sets, development and validation of outcome instruments

We confirm that we have read this submission and believe that we have an appropriate level of expertise to confirm that it is of an acceptable scientific standard.

# Reviewer report: Reviewer response for version 1

**DOI:** 10.5256/f1000research.152547.r243145

**Published:** 2024-02-28

**Authors:** Kim Thomas, Emma Campbell

**Affiliations:** 1 Centre of Evidence Based Dermatology, School of Medicine, University of Nottingham, Nottingham, UK

Uptake of core outcome sets by clinical trialists in China: a protocol

 Thank you for asking for a review of this interesting study protocol. Overall, the protocol is well written.

 The authors state that the two aims of this protocol are to:

1. Determine whether ongoing and completed COS are relevant to the disease burden in China.
2. Find out whether clinical trialists in China use published COS in their trials.

They have identified the top 25 disease categories in China, based on the number of disability-adjusted life years (DALYs). They plan to search the COMET database to find ongoing and published Core Outcome Sets (COS) that have been added since 2012, that are relevant to these 25 disease categories.

 They will also search the Chinese Clinical Trial Registry to identify clinical trials (registered 2021-2022) in the 25 disease categories that list outcomes that match the outcomes in the disease-specific COS. This will give an indication of whether ongoing and completed COS are relevant to the disease burden in China.

 For further analysis, individual clinical trials will be analysed to see whether an appropriate COS exists for the disease in question. Each trial also has to meet the inclusion criteria of there being at least 40 clinical trials that match to each specific COS. Lead investigators of the selected clinical trials will then be surveyed, and some will be interviewed, on their views of COS.

 This is an ambitious study that will provide an interesting insight into COS uptake in China. It is actually three studies in one:

1.
  1. Mapping of burden of disease in China to published COS.
  2. Exploring uptake of COS in trials registered on the Chinese clinical trials registry.
  3. Mixed methods study to establish awareness, barriers and facilitators to COS uptake (including both an initial survey and subsequent interview study).

It might have been helpful to describe these three aspects separately, with specific aims, objectives and methods for each.  Not all of the studies aims and objectives are clearly articulated in the existing two aims.

 The authors suggest that specific COS may be required to evaluate interventions testing Chinese Medicine and that uptake of COS developed in China might be higher than uptake of COS developed internationally. I am a little worried that such a stance goes against the very essence of what COS initiatives are trying to do.  Namely to promote a single unified approach to evaluating outcomes in a given condition throughout the world.  I am not yet convinced that a COS specific to Chinese Medicine is helpful or required.

 The approach of focussing on the top 25 diseases by burden of disease in China is interesting and should help to focus the study, although this still represents a lot of work.  It is not clear why only COS published since 2012 are included. If a disease category has a gold-standard COS that was published pre-2012 (e.g the work of the OMERACT group), this protocol would count the disease category as having no COS, which seems a shame.

 It is proposed that the COS identified through a search of the COMET database will be evaluated for quality.  Whilst this is a laudable ambition, there was very little detail provided as to exactly how this would be done and what the cut-off for meeting the quality standard might be.  Rather than excluding COS based on their quality status, I wonder if it would be more useful to include all identified COS, and to report the quality ratings descriptively.  At least this information would then be in the public domain and could avoid wasted effort of people repeating these quality assessments.

 One of the specified inclusion criteria for study 2 (uptake of COS in clinical trials and studies) required that there be “not less than 40 trials for a given condition”. It might be helpful to add an explanation as to why this was included.  If trials are only included in cases where at least 40 of them match a specific COS, the authors are not capturing up to 39 trials per disease category.

 Similarly, in the section describing data extraction, it might be helpful to signpost to the fact that study 3 will require the contact details of clinical trialist who have been identified in study 2.  Otherwise, the reader is left wondering why this information was being extracted from the registry data.

 In the data extraction section, it states that ‘data will be retrieved from previous COMET systematic reviews’ but it is not clear how this information will be used in the protocol.

 We have suggested a few minor wording amendments to improve readability:

- Background

Suggest amend to: “There are some benefits to using COS in clinical trials, which include improving the consistency of outcome reporting, increasing the relevance of outcomes measured to decision-makers, and helping to identify potential selective outcome reporting bias.” 

- Methods

Confusing typo in survey section says ‘A survey will be sent to clinical trialists who will conduct a clinical trial’ – should this be ‘A survey will be sent to clinical trialists who have conducted a clinical trial’?

 In the survey, there is a question about whether the respondent is a working in Traditional Chinese Medicine, Integrative Medicine or Western Medicine. Integrative Medicine is not mentioned anywhere in the protocol.

 We wish the authors good luck with conducting this study and look forward to reading the results.

Is the study design appropriate for the research question?

Yes

Is the rationale for, and objectives of, the study clearly described?

Partly

Are sufficient details of the methods provided to allow replication by others?

Partly

Are the datasets clearly presented in a useable and accessible format?

Not applicable

Reviewer Expertise:

Applied health research, core outcome sets, development and validation of outcome instruments

We confirm that we have read this submission and believe that we have an appropriate level of expertise to confirm that it is of an acceptable scientific standard, however we have significant reservations, as outlined above.

# Response

**Published:** 2024-03-18

**Authors:** Ruijin Qiu

**Affiliations:** Beijing University of Chinese Medicine Affiliated Dongzhimen Hospital, Beijing, China

**Reviewer comment 1**. This is an ambitious study that will provide an interesting insight into COS uptake in China. It is actually three studies in one:

 Mapping of burden of disease in China to published COS.

 Exploring uptake of COS in trials registered on the Chinese clinical trials registry.

 Mixed methods study to establish awareness, barriers and facilitators to COS uptake (including both an initial survey and subsequent interview study).

 It might have been helpful to describe these three aspects separately, with specific aims, objectives and methods for each.  Not all of the studies aims and objectives are clearly articulated in the existing two aims.

**Author response 1. **The three aims were listed in the final paragraph of the Background section, and the methods for each are described in separate subsections of the Methods section.

**Reviewer comment 2**. The authors suggest that specific COS may be required to evaluate interventions testing Chinese Medicine and that uptake of COS developed in China might be higher than uptake of COS developed internationally. I am a little worried that such a stance goes against the very essence of what COS initiatives are trying to do.  Namely to promote a single unified approach to evaluating outcomes in a given condition throughout the world.  I am not yet convinced that a COS specific to Chinese Medicine is helpful or required.

**Author response 2. **We did not intend to suggest that, and have included the rationale for including a COS developed by Chinese researchers in the protocol.

**Reviewer comment 3**. The approach of focussing on the top 25 diseases by burden of disease in China is interesting and should help to focus the study, although this still represents a lot of work.  It is not clear why only COS published since 2012 are included. If a disease category has a gold-standard COS that was published pre-2012 (e.g the work of the OMERACT group), this protocol would count the disease category as having no COS, which seems a shame.

**Author response 3. **All COS will be analyzed in the mapping of burden of disease in China to COS. A COS published since 2012 would be preferred to one published before 2012 for the analysis of COS uptake if one meets the eligibility criteria. It has been clarified in the methods.

**Reviewer comment 4. **It is proposed that the COS identified through a search of the COMET database will be evaluated for quality.  Whilst this is a laudable ambition, there was very little detail provided as to exactly how this would be done and what the cut-off for meeting the quality standard might be.  Rather than excluding COS based on their quality status, I wonder if it would be more useful to include all identified COS, and to report the quality ratings descriptively.  At least this information would then be in the public domain and could avoid wasted effort of people repeating these quality assessments.

**Author response 4. **We have clarified that we will use patient participation in COS development as a marker of methodological quality, with the rationale provided.

**Reviewer comment 5. **One of the specified inclusion criteria for study 2 (uptake of COS in clinical trials and studies) required that there be “not less than 40 trials for a given condition”. It might be helpful to add an explanation as to why this was included.  If trials are only included in cases where at least 40 of them match a specific COS, the authors are not capturing up to 39 trials per disease category.

**Author response 5. **The online survey will be sent to trialists who registered clinical trials which are relevant to a specific COS. We anticipate a 50% non-response rate, thus if the number of trials is too small, it will be difficult to obtain a reasonable spread of perspectives from trialists about the use of the relevant COS. It was felt that responses from 20 trialists would be helpful based on a typical number of participants involved in qualitative research, thus requiring an anticipated 40 trials This rationale has been explained in the methods (2.2.3).

**Reviewer comment 6. **Similarly, in the section describing data extraction, it might be helpful to signpost to the fact that study 3 will require the contact details of clinical trialist who have been identified in study 2.  Otherwise, the reader is left wondering why this information was being extracted from the registry data.

**Author response 6. **This has been explained in 2.2.4.

**Reviewer comment 7. **In the data extraction section, it states that ‘data will be retrieved from previous COMET systematic reviews’ but it is not clear how this information will be used in the protocol.

**Author response 7. **Previous COMET systematic reviews, and ongoing inclusion of published COS by COMET, will be used for details of published COS relevant to this work. Data extraction will include the intended use of recommendations, disease category,  disease name, population characteristics (age, sex), interventions, geographical locations of COS developers, geographical locations of COS participants will be extracted, and whether patients participated. This has been explained in 2.1.4.

**Reviewer comment 8. **We have suggested a few minor wording amendments to improve readability:

 Background

 Suggest amend to: “There are some benefits to using COS in clinical trials, which include improving the consistency of outcome reporting, increasing the relevance of outcomes measured to decision-makers, and helping to identify potential selective outcome reporting bias.”

 Methods

 Confusing typo in survey section says ‘A survey will be sent to clinical trialists who will conduct a clinical trial’ – should this be ‘A survey will be sent to clinical trialists who have conducted a clinical trial’?

**Author response 8. **We have made these changes in the manuscript.

**Reviewer comment 9. **In the survey, there is a question about whether the respondent is a working in Traditional Chinese Medicine, Integrative Medicine or Western Medicine. Integrative Medicine is not mentioned anywhere in the protocol.

**Author response 9.** The text has been amended.

# Reviewer report: Reviewer response for version 1

**DOI:** 10.5256/f1000research.152547.r223685

**Published:** 2023-11-30

**Authors:** Zehuai Wen

**Affiliations:** 1 Key Unit of Methodology in Clinical Research, Guangdong Provincial Hospital of Chinese Medicine, Guangzhou, China

This is an exciting study with expected results. As far as I know, this may be the first survey in this field in China. The protocol was clearly reported in terms of the rationale, purpose, and procedure of the study. 

 In addition to the literature review, the study also designed a survey for trial investigators, which is a commendable survey to understand further the current situation of knowledge and application of COS among clinical trial investigators in China, which is also conducive to the development and promotion of COS. 

 From the structured questions in the survey, it should be possible to analyze why the investigators used or did not use COS, so it is recommended to consider this analysis in the statistical analysis section.

Is the study design appropriate for the research question?

Yes

Is the rationale for, and objectives of, the study clearly described?

Yes

Are sufficient details of the methods provided to allow replication by others?

Yes

Are the datasets clearly presented in a useable and accessible format?

Not applicable

Reviewer Expertise:

Evidence-based medicine research in traditional medicine; clinical epidemiology; COS development

I confirm that I have read this submission and believe that I have an appropriate level of expertise to confirm that it is of an acceptable scientific standard.

# Response

**Published:** 2024-03-18

**Authors:** Ruijin Qiu

**Affiliations:** Beijing University of Chinese Medicine Affiliated Dongzhimen Hospital, Beijing, China

Thank you for your comments and useful feedback. From the structured questions in the survey, trialists who searched COS before registration will be asked about the reason why they did not use the COS; their responses will be analyzed by content analysis.
